# Single cell sequencing data identify distinct B cell and fibroblast populations in stricturing Crohn's disease

**DOI:** 10.1111/jcmm.18344

**Published:** 2024-04-29

**Authors:** David T. Humphreys, Amy Lewis, Belen Pan‐Castillo, Giulio Berti, Charles Mein, Eva Wozniak, Hannah Gordon, Radha Gadhok, Annamaria Minicozzi, Joanna ChinAleong, Roger Feakins, Eleni Giannoulatou, Louisa K. James, Andrew J. Stagg, James Oliver Lindsay, Andrew Silver

**Affiliations:** ^1^ Victor Chang Cardiac Research Institute Sydney New South Wales Australia; ^2^ St Vincent's Clinical School University of New South Wales Sydney New South Wales Australia; ^3^ Centre for Genomics and Child Health, Blizard Institute Barts and The London School of Medicine and Dentistry London UK; ^4^ Genome Centre, Blizard Institute Barts and The London School of Medicine and Dentistry London UK; ^5^ Centre for Immunobiology, Blizard Institute Barts and The London School of Medicine and Dentistry London UK; ^6^ Department of Colorectal Surgery, Division of Surgery and Perioperative Care The Royal London Hospital London UK; ^7^ Department of Histopathology The Royal London Hospital London UK; ^8^ Department of Cellular Pathology Royal Free London NHS Foundation Trust London UK

**Keywords:** B cells, Crohn's disease, fibroblasts, fibrosis, stricturing

## Abstract

Single cell RNA sequencing of human full thickness Crohn's disease (CD) small bowel resection specimens was used to identify potential therapeutic targets for stricturing (S) CD. Using an unbiased approach, 16 cell lineages were assigned within 14,539 sequenced cells from patient‐matched SCD and non‐stricturing (NSCD) preparations. SCD and NSCD contained identical cell types. Amongst immune cells, B cells and plasma cells were selectively increased in SCD samples. B cell subsets suggested formation of tertiary lymphoid tissue in SCD and compared with NSCD there was an increase in IgG, and a decrease in IgA plasma cells, consistent with their potential role in CD fibrosis. Two Lumican‐positive fibroblast subtypes were identified and subclassified based on expression of selectively enriched genes as fibroblast clusters (C) 12 and C9. Cells within these clusters expressed the profibrotic genes *Decorin* (C12) and *JUN* (C9). C9 cells expressed *ACTA2*; ECM genes *COL4A1*, *COL4A2*, *COL15A1*, *COL6A3*, *COL18A1* and *ADAMDEC1*; *LAMB1* and *GREM1*. GO and KEGG Biological terms showed extracellular matrix and stricture organization associated with C12 and C9, and regulation of WNT pathway genes with C9. Trajectory and differential gene analysis of C12 and C9 identified four sub‐clusters. Intra sub‐cluster gene analysis detected 13 co‐regulated gene modules that aligned along predicted pseudotime trajectories. *CXCL14* and *ADAMDEC1* were key markers in module 1. Our findings support further investigation of fibroblast heterogeneity and interactions with local and circulating immune cells at earlier time points in fibrosis progression. Breaking these interactions by targeting one or other population may improve therapeutic management for SCD.

## 1 INTRODUCTION

Chronic inflammation is a significant factor in driving intestinal fibrosis and reciprocal interactions between fibroblasts and inflammatory immune cells are key to the pathogenesis of fibrosis.^1^ Stricture formation occurs in 30–50% of Crohn's disease (CD) patients^2^, ^3^ and stricturing CD (SCD) is linked with high levels of morbidity and healthcare utilization.^4^, ^5^ Fibrosis is a transmural process and current pharmacological treatments, including biologic and small molecule drugs, neither reduce fibrosis nor the requirement for stricture resection,^6^ and stricture recurrence is common.^7^

Fibrosis is characterized by increased deposition of extracellular matrix (ECM) proteins such as Collagen‐I with accompanying thickening of both submucosa and smooth muscle cell layers.^8^ Fibroblasts are the primary source of ECM proteins, and both epithelial‐to‐mesenchymal transition (EMT) and the recruitment of circulating fibrocytes contribute to the intestinal fibroblast pool in SCD.^9^, ^10^, ^11^ The increase in ECM production in SCD is compounded by the reduced expression of enzymes that degrade collagen matrix metalloproteinases (MMPs) and increased expression of tissue inhibitors of MMPs (TIMPs).^1^ The molecular mechanisms underlying SCD are complex, incompletely described and influenced by environmental triggers and genetic susceptibility.^12^, ^13^, ^14^

Here, we report the use of human full thickness CD resection specimens and single cell RNA sequencing (scRNA‐seq) to gain information on the cell types in the SCD and non‐stricturing (NSCD) regions of the CD small bowel.

## 2 METHODS

### 2.1 Patient recruitment and single cell isolation from surgically resected tissue

Appropriate local Ethics Committee approvals (London—City Road and Hampstead Research Ethics Committee; 15/LO/2127) and informed consent were obtained prior to patient recruitment. Human full thickness CD resection specimens from the CD small bowel were washed with Hanks' Balanced Salt solution (HBSS) supplemented with 0.01% Dithiothreitol and then HBSS‐EDTA (1 mM) for 10 min per wash under agitation at 37°C. Finely dissected full thickness tissue was incubated with 20 mL of Dulbecco's modified Eagle's medium (DMEM) (Gibco) supplemented with collagenase type 1A (1 mg/mL, c2674‐500 mg, Sigma) and DNase I (10 units/mL) for 45–60 min under gentle agitation, then filtered through a 100 μm cell strainer and the cell pellet washed twice in PBS. For scRNA‐seq cells were re‐suspended in 1 mL FBS supplemented with 10% DMSO and cryostored in line with the recommended 10x genomic protocol for Fresh Frozen Human Peripheral Blood Mononuclear Cells.

### 2.2 Single cell RNA sequencing

SCD and NSCD tissue was collected and processed independently (*n* = 8 in total; SCD, 4 and NSCD 4 samples) as above. Before library preparation, cell suspensions were thawed, washed (PBS BSA 0.04%), filtered and cell viability assessed; a viability cut‐off of >70% was set for each sample to proceed. ScRNA‐seq libraries were generated using the Chromium™ 3’ Library and Gel Bead Kit v3 (PN‐1000092) (10X Genomics, California, USA). Final libraries were run on three NextSeq500 High‐output v2.5 150‐cycle kits (Illumina, CA) with a 26[8]98 cycle configuration to generate 50,000 reads per cell (10X Genomics recommendation).

Sequence reads were aligned with cellranger (v3.1.0; 10x genomics). Imported single cell gene counts were analysed using R package Seurat (v4). A data set was prepared per sample and each was normalized (function “NormalizeData”) followed by the identification of variable features (function “FindVariableFeatures” using parameters “vst”method and nfeatures = 2000). The fraction of mitochondrial genes was calculated for quality assessment (function “PercentageFeatureSet”). Data sets were integrated by selecting integration features and anchors and then using the function “IntegrateData.” Integration data were then scaled (function “ScaleData”) and batch effects regressed out by incorporating patient ID. Principle component analysis (functions “RunPCA” and “FindNeighbors”) was used for graph based clustering at a resolution of 0.3 and Uniform Manifold Approximation (UMAP) and *t*‐SNE dimensionality reduction was then computed (functions “RunUMAP”and “RunTSNE”). Batch correction was performed using Harmony by integrating the patient ID variable.^15^ Fibroblast and B clusters were identified by visualizing cell markers (e.g., Lumican (LUM) and CD83 respectively) before being organized as independent sub‐sets and analysed further. Monocle3 was used to subcluster and analyse pseudotime trajectories of B cell and fibroblast lineages.^16^ Other cell clusters were identified using scAnnotatR,^17^ as well as cross referencing markers provided in the single‐cell type transcriptomics map and protein atlas of human tissues.^17^, ^18^, ^19^

Cross validation analysis of fibroblast cells (CD data set to the fibroblast atlas) used custom R scripts and marker gene identification (Seurat FindMarkers function) from both data sets. Tables of overlapping marker genes were assembled with the respective fold change metrics. Data imported into Cytoscape and StringDB networks were prepared using Omics visualizer app.^20^

## 3 RESULTS

### 3.1 Stricture‐specific cell types in stricturing Crohn's disease

**FIGURE 1:**
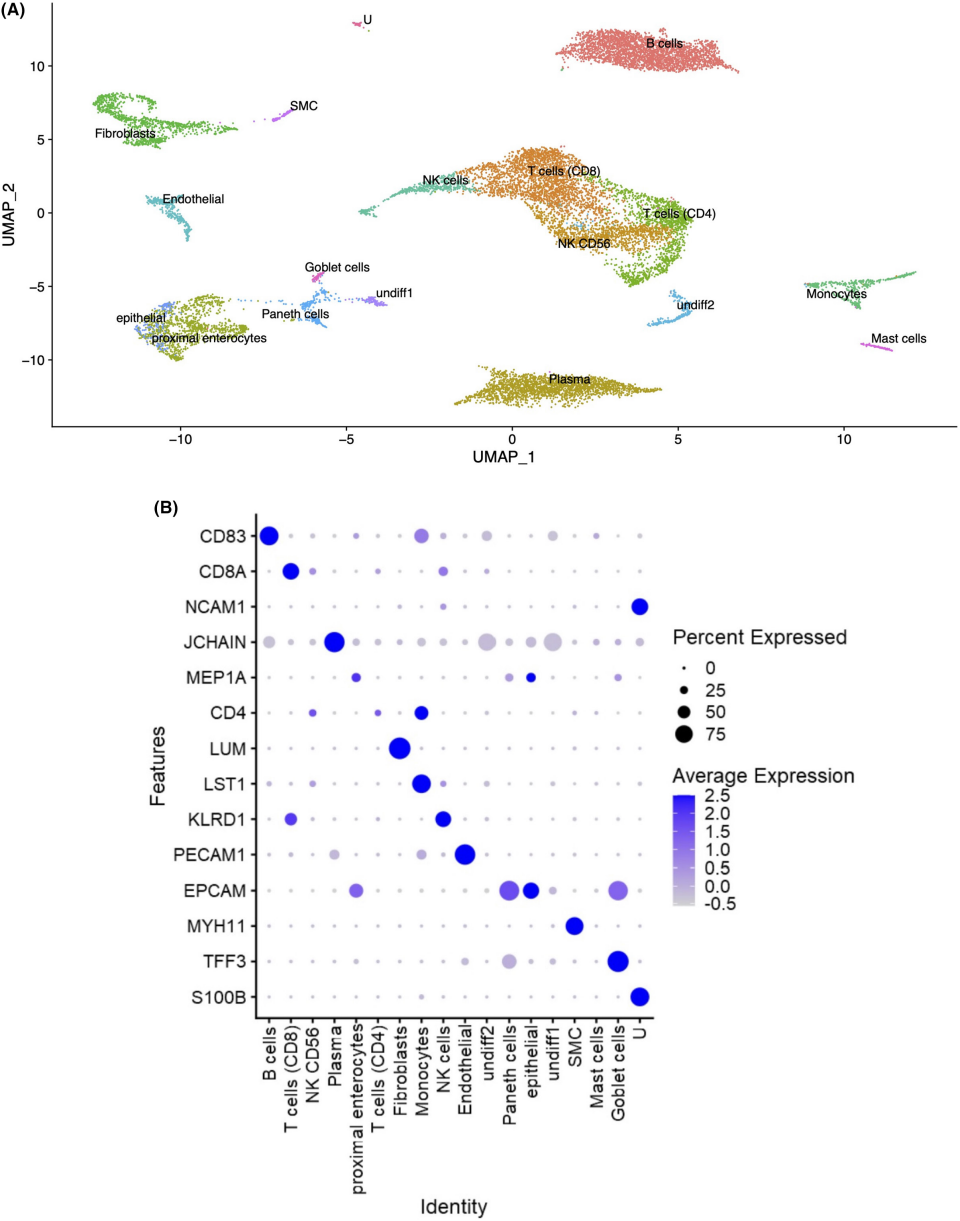
Cellular profile of stricturing Crohn's disease (SCD). ScRNA‐seq was performed on cells from full‐thickness tissue surgically resected specimens from stricturing (SCD; *n* = 4) and non‐stricturing (NSCD; *n* = 4) ileum of CD patients (*n* = 5). Three patients provided both SCD and NSCD specimens. No prior cell selection was applied (e.g., FACs sorting) and all cells (*n* = 14,539) were sequenced. (A) Uniform Manifold Approximation (UMAP) identified 24 clusters assigned to 16 definable cell lineages (Figure S1A). Undifferentiated cells (Undiff1/2) contained markers of undifferentiated cells as defined by protein atlas webtool,^20^ and expressed TOP2A and MKI67 unlike any other cell type. Unknown (U) had no identifiable gene marker linked to any cell type. (B) Bubble plot shows levels of expression of key markers for different cell types identified from published data sets.

**FIGURE 2:**
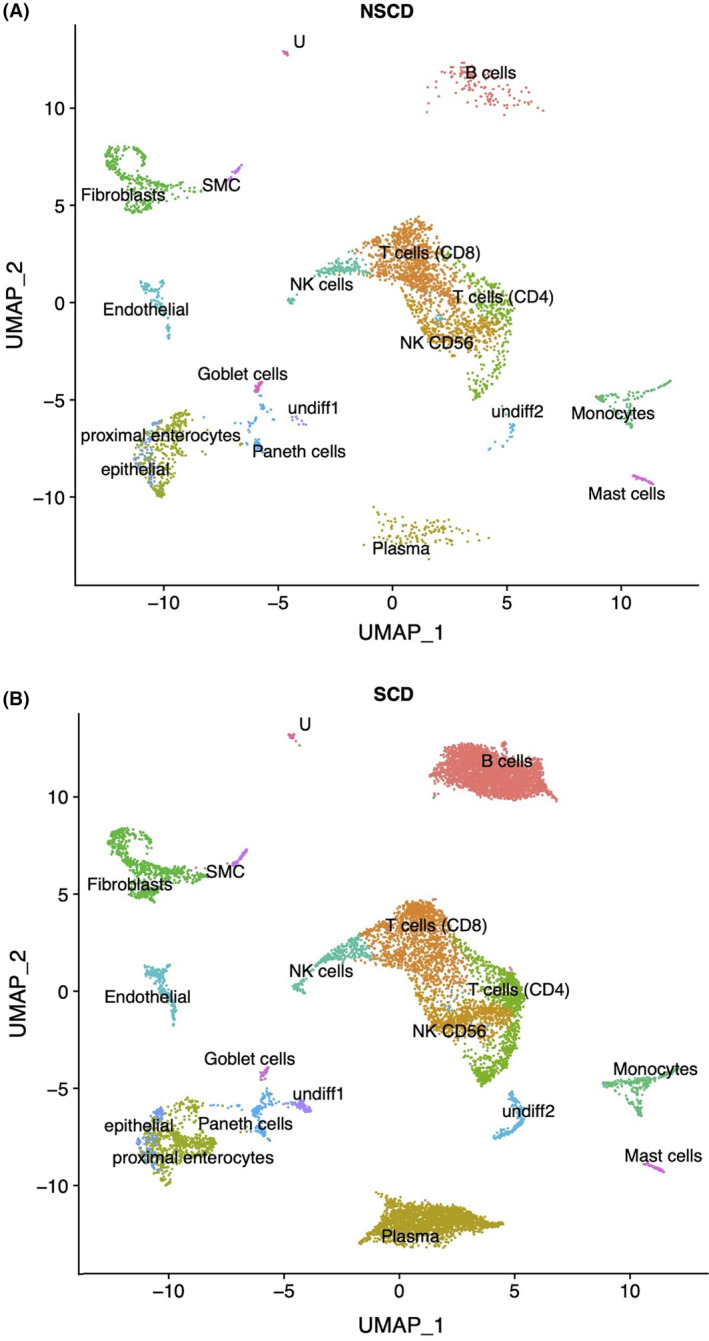
(A) Stricturing (SCD) and (B) non‐stricturing (NSCD) small bowel tissue comprise the same cell types but proportions differ markedly for B cells and plasma cells. A UMAP of NSCD and SCD preparations indicate they are comprised of the same cell types. B cells and plasma cells showed the most marked increased in frequency in SCD compared to NSCD (see Table 2).

We performed scRNA‐seq of cells isolated from full‐thickness tissue surgically resected from the strictured ileum of CD patients (*n* = 4) and non‐strictured CD controls (NSCD *n* = 4); 3/4 were patient matched (Table 1). No prior selection was applied (e.g., FACS) to avoid cell type bias on sequencing. Instead, all cells were sequenced (total of 14,539 cells), integrated and batch corrected.^15^ UMAP visualized the complete integrated data set. This identified 24 clusters which were assigned to 16 definable cell lineages based on cell markers obtained from the published literature (Figure 1).^20^, ^21^ NSCD and SCD preparations contained the same cell types (Figure 2; Table 2) and the same proportions of endothelial and fibroblasts, indicating that phenotype did not impact cell release (Table 2; Figure S1A,B).

**TABLE 1:** Clinical characteristics of Crohn's disease cohort.

Sample ID	Age	Gender	Age at diagnosis	Montreal	Active disease	Medications at surgery	NSCD	SCD	Site
19RLH037	40	M	23	A2L1L4B3	Yes	Infliximab Thiopurine	Yes	Yes	Ileum
20RLH009	27	M	11	A1L3L4B3	Yes	Ustekinumab	Yes	Yes	Ileum
20RLH018	51	M	48	A3L3B2	Yes	Azathioprine	No	Yes	Ileal/caecal
20RLH019	34	M	4	A1L3B3	Yes	Ustekinumab	Yes	No	Ileum
21RLH‐4521	21	M	18	A2L3B2	Yes	Ustekinumab	Yes	Yes	Ileum

*Note*: All cases included in our study were “sporadic” Crohn's disease (CD) with no cases of very early onset CD. This is evidenced by the fact that the disease duration at the time of surgery for the 2 paediatric diagnoses was 16 and 30 years. Several patients had a history of penetrating disease (Montreal B3), but resection specimens used in this analysis were harvested by a GI pathologist to ensure that they derived from fibrotic structures (SCD) or non strictured (NSCD) segments away from the site of any penetration.

**TABLE 2:** B cells and plasma cells showed the most marked increased in frequency in stricturing Crohn's disease (SCD) compared to non‐strictured (NSCD).

Cell type	NSCD	SCD
B cells	123 (3.6)	2846 (25.6)
T cells (CD8)	969 (28.4)	1380 (12.4)
T cells (CD4)	760 (22.2)	1736 (15.6)
Plasma cells	127 (3.7)	2076 (18.7)
Proximal enterocytes	345 (10.1)	727 (6.5)
Fibroblasts	347 (10.1)	694 (6.2)
Monocytes	125 (3.7)	275 (2.5)
NK cells	170 (5)	331 (3.0)
Endothelial	123 (3.6)	242 (2.2)
Undiff2	34 (1)	241 (2.2)
Paneth cells	84 (2.5)	165 (1.5)
Epithelial	66 (1.9)	103 (0.9)
Undiff1	7 (0.2)	101 (0.9)
SMC	34 (1)	71 (0.6)
Mast cells	44 (1.3)	59 (0.5)
Goblet cells	39 (1.1)	31 (0.3)
U	20 (0.6)	44 (0.4)
Total	3417	11,122

*Note*: Numbers and frequency (%) of cells identified from NSCD and SCD small bowel tissue.

### 3.2 Stricturing Crohn's disease is associated with selective increases in B cells and IgG plasma cells

**FIGURE 3:**
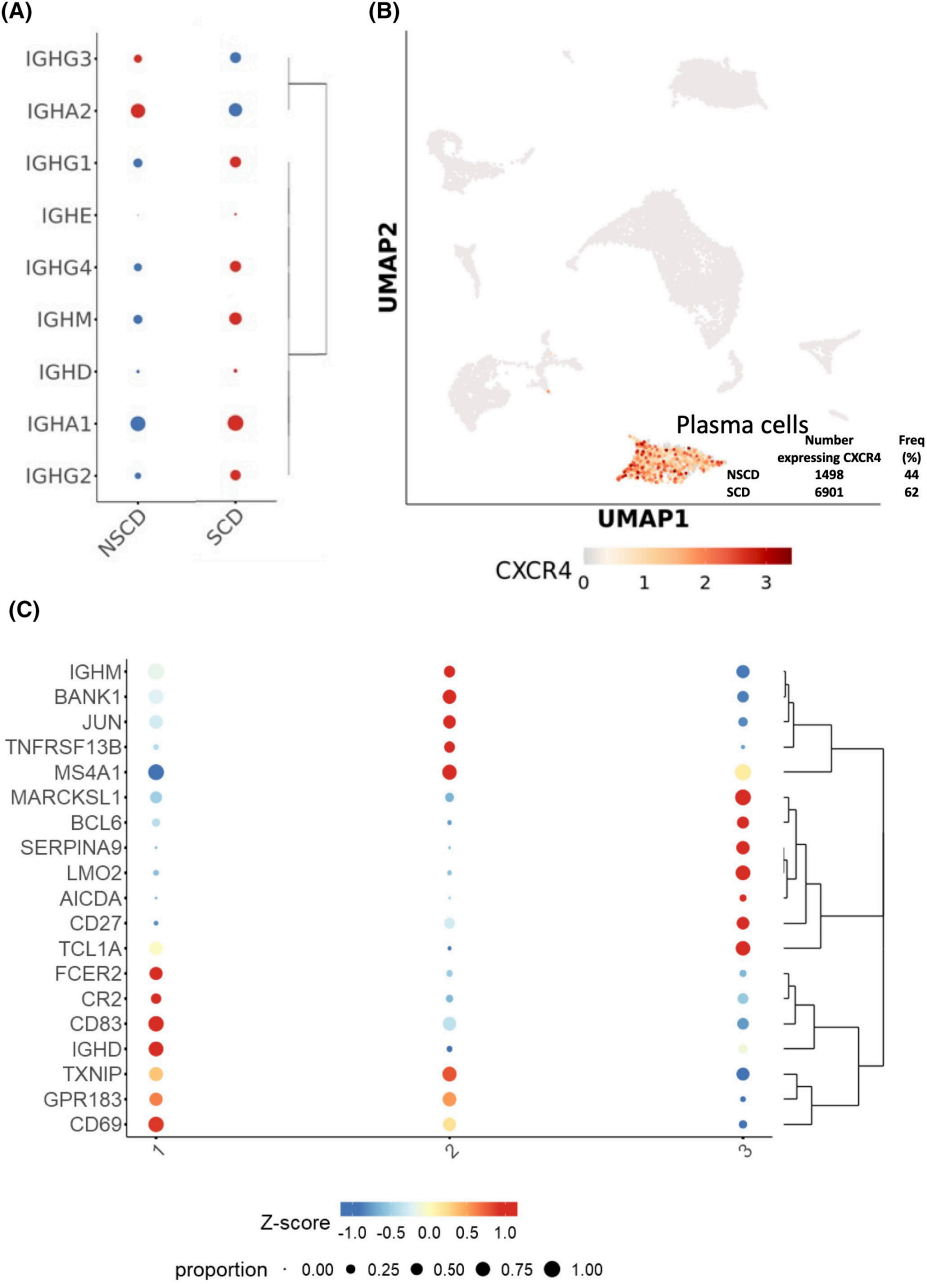
Analysis of B cells and plasma cells in stricturing (SCD) and non‐stricturing (NSCD) small bowel tissue. (A) Proportions of antibody subclasses expressed by plasma cells in SCD and NSCD tissues. (B) Expression of *CXCR4* in plasma cells. (C) Sub‐clustering of B cells identified three distinct clusters consistent with naïve (1), memory (2) and germinal centre (3) subsets.

Fibroblast function under inflammatory conditions is shaped by interaction with immune cells.^1^ Amongst immune cell types, B cells and plasma cells were most markedly increased in frequency in SCD compared to NSCD (Figure 2; Table 2). As well as differences in absolute numbers of plasma cells, there were differences in the distribution of antibody subclasses produced by these cells. The proportion of IgG subclasses was increased in SCD, whereas IgA2, was increased in NSCD (Figure 3A). *CXCR4*, which is reported to facilitate plasma cell homing to inflamed tissue was expressed by 62% of plasma cells in SCD versus 44% in NSCD (Figure 3B).^22^ Additional sub‐clustering of B cells identified a population of naïve B cells (Figure 3C, cluster 1), expressing *IGHD* and *IGHM*; a cluster expressing genes consistent with a memory B cell phenotype (Figure 3C, cluster 2); and a small subset of B cells expressing germinal centre markers including *BCL6* and *AICDA* (Figure 3C, cluster 3), the latter of which were almost exclusively found in SCD but not NSCD.

### 3.3 Identification of two fibroblast subtypes

**FIGURE 4:**
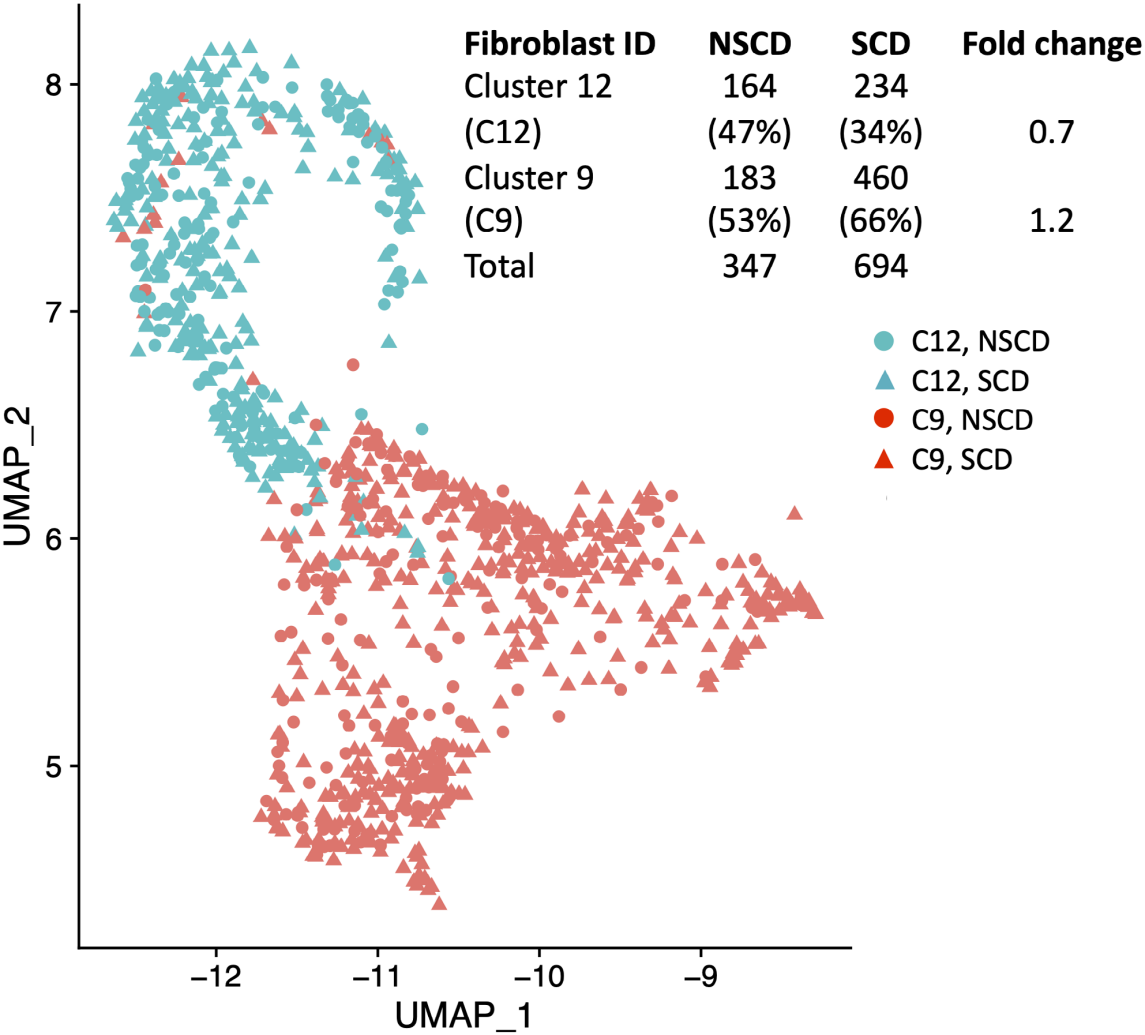
Identification of the C12 and C9 fibroblast subtypes in stricturing and non‐stricturing small bowel tissue. UMAP of fibroblasts identifying cluster 12 (all C12, blue symbols) and cluster 9 (all C9, red symbols) from the integrated stricturing (SCD; triangles) and non‐stricturing (NSCD, dots) scRNA data set. Insert shows a comparison of the numbers of LUM+ve C12 and C9 clusters in resected small bowel from both NSCD and SCD tissue. The percentage of each cluster in either the NSCD or SCD data set is given in parenthesis. The fold change in C12 and C9 fibroblasts in NSCD and SCD is shown as SCD/ NSCD.

LUM is an ECM‐secreted proteoglycan known to regulate collagen fibrogenesis and two LUM+ve fibroblast subtypes were identified from our scRNA‐seq data (Figure 1A,B). These were further subclassified as two clusters (fibroblast cluster 12 (C12) and fibroblast cluster 9 (C9)) based on expression of selectively enriched genes (fold change >1.5 and adjusted *p* ≤ 0.001; Figure 4; Table S1). Ranking these genes according to the numbers of cells in which they were expressed demonstrated that the highest proportion of cells within the two clusters expressed the profibrotic genes *Decorin* (*DCN*) for C12 and *JUN* for C9 (Table S1). Based on prior studies we considered the expression of key genes (collagen genes or potential markers of fibrosis) involved in the fibrotic process (Table S1). Examination of the gene list independent of position within Table S1, but relevant to the fibrotic process, revealed increased expression of: a marker of myofibroblasts, *ACTA2*; the ECM collagen genes, *COL4A1*, *COL4A2*, *COL15A1*, *COL6A3*, *COL18A1*; a marker of a distinct fibroblast population in fibrosis, *ADAMDEC1*; and *LAMB 1* and *GREM1*, both reported to be involved fibrosis (C9 compared to C12; *p*‐value adjusted < 9.0E‐05; Table S1).^23^, ^24^, ^25^

### 3.4 Extracellular matrix, stricture organization and regulation of WNT pathway genes are associated with fibroblast clusters

**FIGURE 5:**
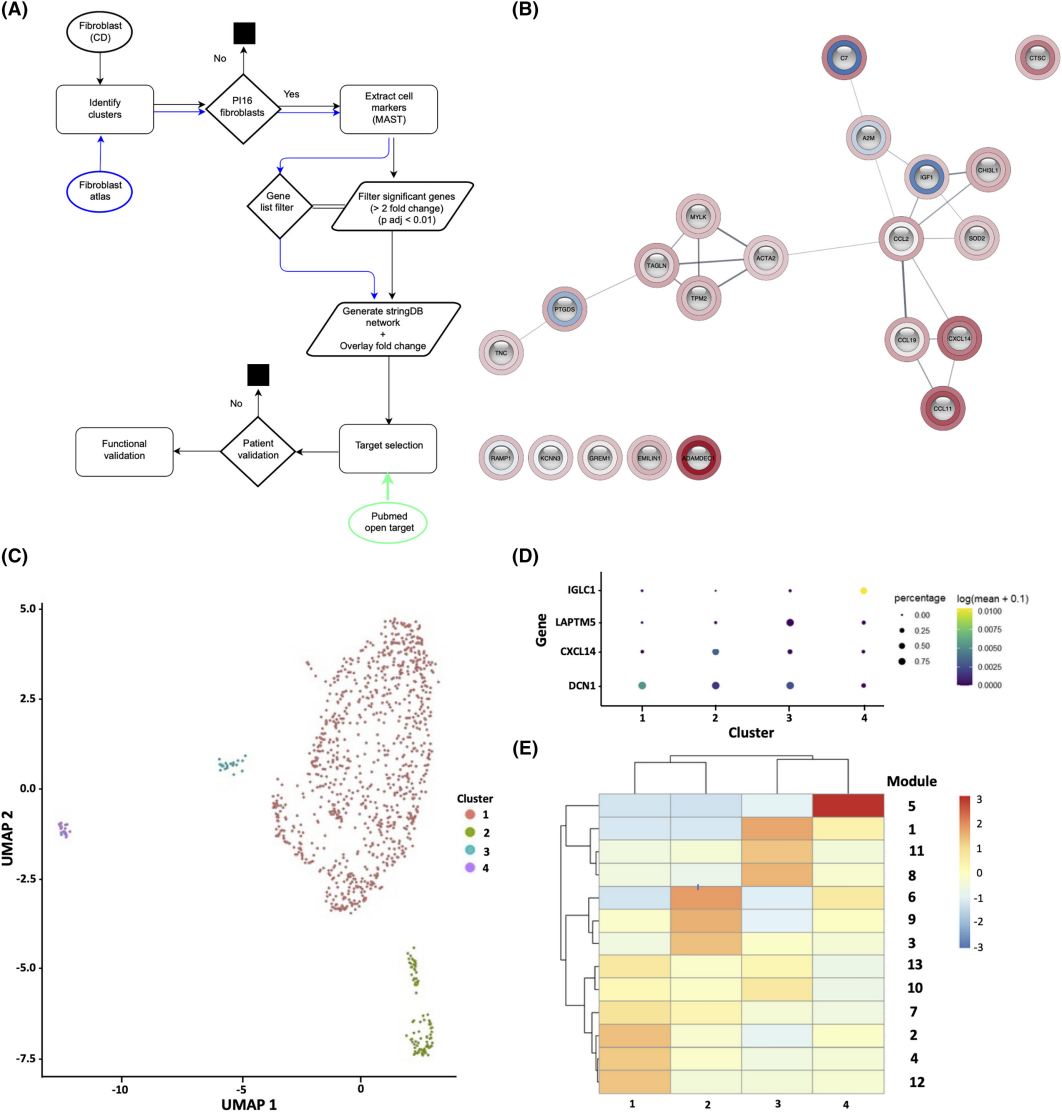
Workflow and stringdb network assembly, trajectory and differential gene analysis for fibroblast populations and clusters. (A) Schematic of scRNA‐seq workflow, incorporating stringdb network assembly for C9‐like fibroblasts and target validation workflow. (B) Stringdb network for C9‐like SCD fibroblasts. (C) Four clusters resolved from differentiation trajectories of the two fibroblast populations were analysed using Monocle3.^16^ (D) Identification of the most significant gene marker for each cluster using differential gene analysis. (E) Co‐regulated gene modules identified from predicted pseudotime trajectories using Monocle3.^16^ Each module contains multiple genes that have similar expression patterns between cell neighbours (i.e. intracluster gene analysis). Gene members of each gene module are listed in Figure S3.

The GO and KEGG Biological terms associated with the fibroblast cluster C12 and C9 gene sets included ECM and stricture organization (both clusters; GO terms), and positive and negative regulation of *WNT* pathway genes (C9; GO terms) and the IL‐17 signalling pathway (C9 KEGG terms) (Figure S2A–H). The fibroblast subtypes corresponded to those identified previously by scRNA‐seq in ulcerative colitis (UC) and other fibrotic diseases.^23^, ^26^ C9 fibroblast markers (Table S2) were validated independently by comparing with PI16 fibroblasts in the perturbed human fibroblast atlas (fibroXplorer database^26^; *n* = 3604 fibroblasts; Figure 5A,B). Single cell trajectory analysis was performed on all fibroblasts to identify possible differentiation states^27^ and from this, four sub‐clusters were identified (Figure 5C). Differential gene analysis identified gene markers for each sub‐cluster and intra sub‐cluster gene analysis identified co‐regulated gene modules that aligned along predicted pseudotime trajectories (Figure 5D,E). The modules represent groups of cells with gene expression profiles that are sufficiently unique to allow their separation into individual modules with a unique identify. Some genes may be included in more than one module. The gene members of each of the 13 gene modules are shown in Figure S3. The top two markers of module 1 were *CXCL14* and *ADAMDEC1* (Figure S3), previously identified to be markers in a distinct fibroblast population in UC and other fibrotic diseases,^23^ indicating that sub‐cluster 1 are the same *ADAMDEC1*‐like fibroblasts.

## 4 DISCUSSION

We have, for the first time, generated sequencing data from all the cell types isolated from resected full‐thickness SCD and NSCD intestinal specimens from stricturing CD patients. Access to well phenotyped, fresh surgical specimens, rather than the more readily accessible mucosal biopsies, allowed sampling from the deeper layers of the bowel that contribute to CD fibrosis. This underlines the clinical importance of our dataset. In contrast to studies in other organ systems that have used enrichment, we determined natural groupings from an atlas of 14,539 sequenced cells. This identified fibroblasts and immune cell populations in the gut without any potential bias associated with pre‐selection. The availability of human IBD datasets, such as fibroXplorer,^26^ supported independent validation.

The numbers of plasma and B cells were increased in SCD tissue to a much greater degree than other immune cell populations. The differential role of IgA1 and IgA2 in the intestine is poorly understood. Cells secreting IgA1 usually predominate in the small intestine and those secreting IgA2 are increased in the colon. IgA2 is more resistant to proteases and thus may persist longer, with an increased effect on the microbiota in the gut lumen when secreted. The altered pattern of expression of IgA1 and 2 in strictured versus non‐strictured tissue might reflect a change in the production of factors controlling isotype switching perhaps as the result of altered microbial status in the strictures versus normal gut tissue. An Imbalance in the gut flora has been reported to possibly promote the mucosa of the terminal ileum to increased IgA production and thereby expand the proportion of IgA‐coated bacteria in Diarrhoea‐Predominant Irritable Bowel Syndrome.^28^ Activating IgA class switching may serve to regulate inflammation and subsequent pathologies,^28^ and it remains to be seen whether this includes intestinal fibrosis.

Several studies have recently identified a role for B cells in the pathogenesis of UC,^22^, ^29^, ^30^ but their contribution to CD remains poorly understood. There was a shift towards plasma cells expressing IgG subclasses, and away from IgA, in SCD. IgG drives inflammation in UC by activating innate immune cells via interactions with Fc receptors and the consequent promotion of inflammatory Th17 responses.^30^ In UC, IgG plasma cells infiltrate the inflamed intestine via CXCR4,^22^ and this chemokine receptor was expressed by many plasma cells in our CD study. IgG plasma cells expressing *CXCR4* were enriched in SCD compared with NSCD samples. Reclustering of B cells revealed naïve, memory and germinal centre B cell populations, the latter of which were almost exclusively found in SCD. These finding are consistent with the formation of organized tertiary lymphoid tissue (TLT) in SCD.^31^ TLT formation is a feature of CD,^32^ but its causal contribution to the inflammatory process or to fibrosis is currently poorly understood. In other inflammatory contexts, B cells have been shown to interact directly with fibroblasts to influence the fibrotic process in inflammation,^33^ raising the possibility that a similar process contributes to fibrosis in CD.

The observed increases in immune cells, both proportionally and in number, in the strictured compared to non‐strictured tissue is probably not surprising given the role of inflammation in the fibrotic process. However, more interesting are the selective changes observed such as the discriminating increase in B cells and the changes observed in the composition of this B cell population. Collectively these observations suggest that B cells and/or IgG may play a significant role in the fibrotic process associated with development and maintenance of strictured regions. The large increase in B cells (and plasma cells) may partially mask smaller increases in populations of other cell types throughout the gut that could contribute potentially to the fibrotic process. Furthermore, we know that recurrent stricture development is common after surgical resection.^7^

Tertiary lymphoid structures (TLS) are a known histological feature of CD. Nevertheless, it remains to be determined if they contribute to CD pathogenesis, or conversely represent a protective response. As our analysis used full thickness intestinal tissues it is likely that we captured such structures and cells that might indicate TLS were identified. However, we cannot comment on the spatial location of different cell types because of the dissociation process necessary for scRNA‐seq. To resolve help these issues, work could be undertaken in the future using single cell spatial transcriptomics. Whilst our data might suggest a link with fibrosis the cellular involvement and mechanisms remain unclear.

Lumican is a member of the class II small leucine‐rich proteoglycan superfamily known to be involved in fibroblast function and organization of collagen fibrils in the ECM.^34^ In this context, LUM is critical for the pathogenesis of several fibrotic diseases, including lung, heart, cornea and liver,^35^, ^36^, ^37^, ^38^, ^39^ and may promote early fibrotic responses to injury.^35^ LUM is also known to be involved in inflammation,^40^ and immune cell recruitment after injury.^41^ Lumican secretion from TNF‐α stimulated fibroblasts can promote differentiation of monocytes to fibroblasts.^42^

We have identified two LUM+ve fibroblast subtypes, which were further subclassified as two clusters, C12 and C9. Their analysis highlighted the expression of profibrotic genes *DCN* and *JUN* (C12 and C9, respectively): DCN modulates TGFβ‐driven fibrosis in a number of organs and JUN induction drives murine pulmonary fibrosis.^43^, ^44^, ^45^ In addition, a number of genes were identified that are involved in the fibrotic process. Cells in the C9 cluster express ACTA2, the marker of myofibroblasts. Increased production of ECM proteins, such as collagen, is a key feature in the mechanism for development of fibrotic strictures and is part of the of wound healing response to inflammation. Accordingly, we identified *COL4A1*, *COL4A2*, *COL15A1*, *COL6A3*, *COL18A1* expression in C9 fibroblasts. These cells also expressed *ADAMDEC1*, previously identified to be a marker in a distinct population in UC and other fibrotic diseases.^23^ *LAMB1* and *GREM1* were also found to be expressed. Interestingly, *LAMB1* and *COL4A2* are potential markers of fibrosis progression in liver and GREM1, a highly conserved member of the TGFβ superfamily, is involved in fibrosis across multiple organ systems.^24^, ^25^ It has been suggested that Lumican‐ inhibiting drugs could be used to modulate fibrosis.^42^ In this context, our finding of two LUM+ve fibroblast subtypes and the expression of profibrotic genes by C12 and C9 cluster cells may support the identification of suitable drug targets.

Interestingly, positive and negative regulation of WNT pathway genes were found to be associated with the C9 fibroblast cluster in SCD. Fibrosis has been associated with WNT pathway activation in a number of organs such as the lung, liver, kidney, and skin,^46^ and in the development of aggressive complications in inflammatory bowel disease (IBD) patients, notably IBD‐associated colorectal cancer,^47^ and formation of intestinal fistulae.^48^ Recently we identified the importance of cross‐talk between WNT and TGFβ signalling pathways in CD intestinal fibrosis.^49^ The results presented here for C9 cluster fibroblasts are in accordance with this prior study and support further the potential use of small‐molecule inhibitors of WNT signalling in treating intestinal fibrosis in CD by targeting particular fibroblast clusters identified by scRNA‐seq.

Although IL‐17 is considered to play a role in fibrosis in some organs, its role in intestinal fibrosis in human CD is unclear.^50^ A putative role for IL‐17 signalling in CD fibrosis is supported by the finding that an anti‐IL‐17 antibody can diminish colitis and fibrosis in the TNBS murine model through downregulation of collagen expression and pro‐fibrogenic cytokines such as IL‐1β, TGF‐β1, and TNF‐α,^51^ or possibly by reducing EMT.^52^ However, data from two clinical trials did not identify any notable efficacy from blocking IL‐17, which may relate to the importance of the cytokine in intestinal homeostasis including mucosal protection.^50^ Whether blocking of IL‐17 or a family member has a direct anti‐fibrotic effect or is related to an ability to reduce inflammation remains to be established.

In conclusion, our study provides an unbiased reference dataset for SCD versus NCD, which is controlled internally because most of these tissues are matched from the same individual. This enables the internal relationships of different cell types to be compared in a single time window. Our data indicate clearly that therapeutic intervention must come much earlier in CD progression than the timepoint described here to break the apparent interaction between immune cells and fibroblasts. In this context, it would be interesting to consider whether increased immune cells residency in the mucosa and/ or assay of circulating antibodies/ immune cells in blood could indicate fibrotic progression at a much earlier time point.

## AUTHOR CONTRIBUTIONS

**David T. Humphreys:** Data curation (equal); formal analysis (equal); investigation (equal); methodology (equal); resources (equal); writing – review and editing (equal). **Amy Lewis:** Conceptualization (equal); data curation (equal); formal analysis (equal); investigation (equal); methodology (equal); writing – original draft (equal); writing – review and editing (equal). **Belen Pan‐Castillo:** Investigation (equal); methodology (equal). **Giulio Berti:** Investigation (equal); methodology (equal). **Charles Mein:** Investigation (equal); methodology (equal). **Eva Wozniak:** Data curation (equal); investigation (equal); methodology (equal). **Hannah Gordon:** Investigation (equal); resources (equal). **Radha Gadhok:** Investigation (equal); resources (equal). **Annamaria Minicozzi:** Investigation (equal); resources (equal). **Joanna ChinAleong:** Investigation (equal); methodology (equal); resources (equal). **Roger Feakins:** Investigation (equal); methodology (equal); resources (equal). **Eleni Giannoulatou:** Investigation (equal); methodology (equal); resources (equal). **Louisa K. James:** Investigation (equal); writing – review and editing (equal). **Andrew J. Stagg:** Investigation (equal); writing – review and editing (equal). **James Oliver Lindsay:** Conceptualization (equal); resources (equal); supervision (equal); writing – review and editing (equal). **Andrew Silver:** Conceptualization (equal); funding acquisition (lead); project administration (equal); resources (equal); supervision (equal); writing – original draft (lead); writing – review and editing (equal).

## FUNDING INFORMATION

The Silver/Lindsay laboratory is supported by grants from Crohn's and Colitis UK (M2018‐4) and Barts Charity (MGU0399 and G‐001976); D.T.H. and E.G. acknowledge The Victor Chang Cardiac Research Institute Innovation Centre (funded by the New South Wales Government Ministry of Health).

## CONFLICT OF INTEREST STATEMENT

The authors confirm there are no conflicts of interest.

## PREPRINT SERVER

The manuscript was deposited on bioRxiv doi: 10.1101/2023.09.04.556163.

## Supporting information


**Figure S1.** Comparison of cell numbers and types from SCD and NSCD small bowel tissue. (A) Comparison of fibroblast (F) cell numbers with pericytes plus endothelial (P+E) cell numbers from resected SCD and NSCD tissue showed no significant differences suggesting consistent release of cells across both stricturing and non‐stricturing tissue. (B) Comparison of portion of cell types identified for each processed scRNA‐seq specimen where each dot represents a particular resected specimen. The proportion of epithelial cells was as expected and explained by the washing efficiency during tissue collection.

**Figure S2.** Enriched Kegg and GO terms for fibroblast clusters C12 and C9. Top 10 enriched KEGG and GO terms for C12 fibroblasts (A and B respectively), C9 fibroblasts (C and D respectively). Gene signatures from C9 fibroblasts were further analysed by identifying the top 10 enriched KEGG and GO terms of C9 fibroblasts originating from SCD (E and F respectively) or NSCD (G and H respectively) conditions.

**Figure S3.** Gene markers for co‐regulated modules. All gene modules were identified using Monocle3^32^ Note that *CXCL14* and *ADAMDEC1* were the top two markers of module1 (arrowed), and are primarily expressed in sub‐cluster2.

**Table S1.** Gene markers that define fibroblast cell clusters C9 and C12. List of all differentially expressed genes identified using the findmarkers function (MAST algorithm) to compare C9 to C12 fibroblast populations. The table was created using a filtering fold change of >1.5 and adjusted *p*‐values of <0.01. Each table has been ordered on pct.1 and then by average fold change. Genes with known involvement in the fibrotic process and mentioned in the text (collagen genes or potential markers of fibrosis) are highlighted in bold. Whilst these genes are not at the very top of Table S1, they are highly significant (*p*‐value adjusted <9.0E‐05).

**Table S2.** Gene markers that define fibroblast cell clusters C9 and C12 derived from either stricturing Crohns disease (SCD) or non‐stricturing CD (NSCD). List of all differentially expressed genes identified using the findmarkers function (MAST algorithm) to compare cluster (C) 9 SCD, C9 NSCD, C12 SCD and C12 NSCD fibroblast populations. Each table has been ordered on pct.1 and then by average fold change.

## Data Availability

The scRNA‐seq data are available via Array Express accession: https://www.ebi.ac.uk/biostudies/arrayexpress/studies/E‐MTAB‐11792.
