# Supplementary figures and images for: Single cell sequencing data identify distinct B cell and fibroblast populations in stricturing Crohn's disease
Source: J Cell Mol Med. 2024 Apr 29;28(9):e18344. doi: 10.1111/jcmm.18344 (PMC11058334; doi:10.1111/jcmm.18344)

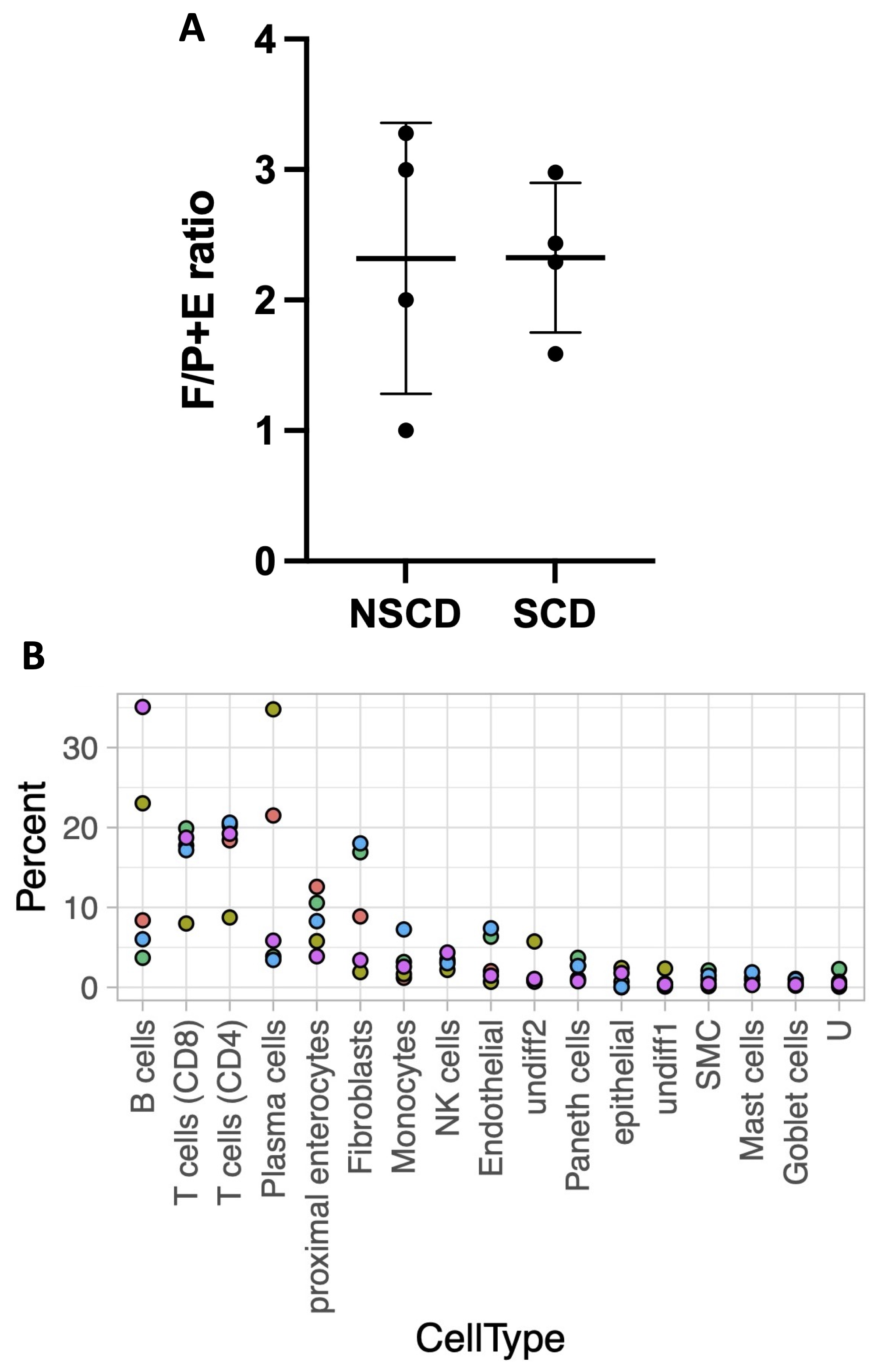

Supplement: Supplementary file 1 — Figure S1. Comparison of cell numbers and types from SCD and NSCD small bowel tissue. (A) Comparison of fibroblast (F) cell numbers with pericytes plus endothelial (P+E) cell numbers from resected SCD and NSCD tissue showed no significant differences suggesting consistent release of cells across both stricturing and non‐stricturing tissue. (B) Comparison of portion of cell types identified for each processed scRNA‐seq specimen where each dot represents a particular resected specimen. The proportion of epithelial cells was as expected and explained by the washing efficiency during tissue collection. [file JCMM-28-e18344-s004.tiff]

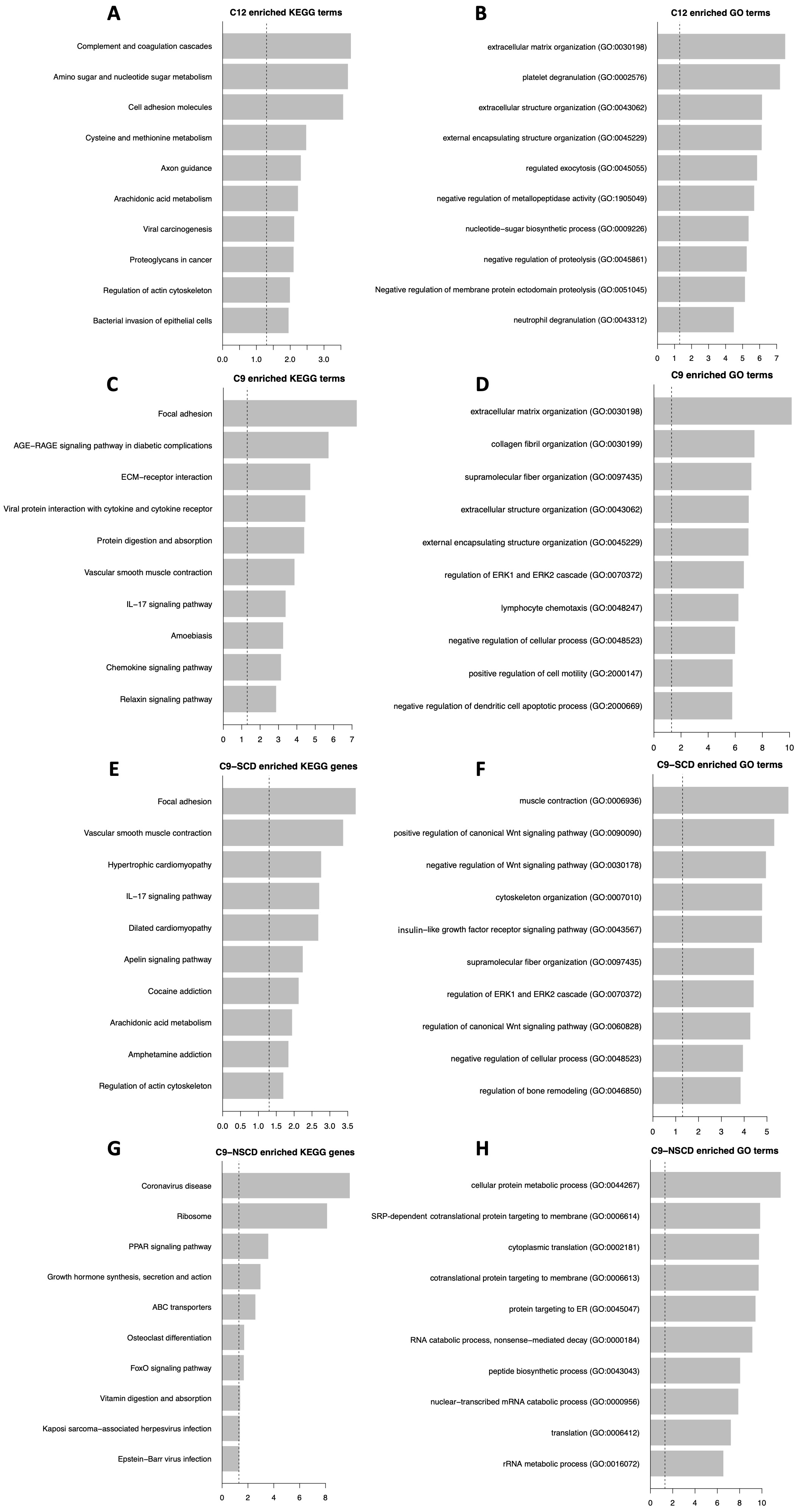

Supplement: Supplementary file 2 — Figure S2. Enriched Kegg and GO terms for fibroblast clusters C12 and C9. Top 10 enriched KEGG and GO terms for C12 fibroblasts (A and B respectively), C9 fibroblasts (C and D respectively). Gene signatures from C9 fibroblasts were further analysed by identifying the top 10 enriched KEGG and GO terms of C9 fibroblasts originating from SCD (E and F respectively) or NSCD (G and H respectively) conditions. [file JCMM-28-e18344-s002.jpg]

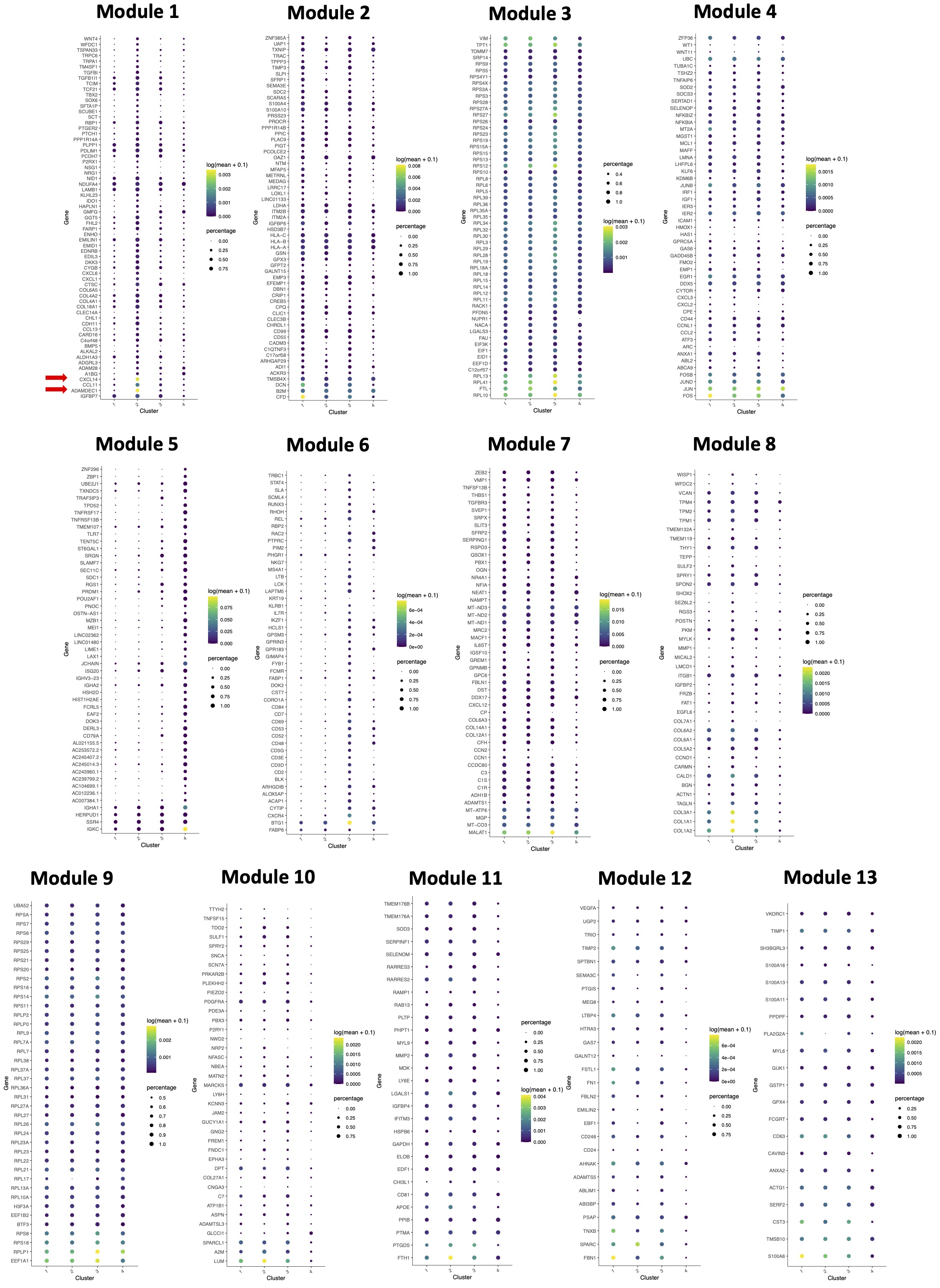

Supplement: Supplementary file 3 — Figure S3. Gene markers for co‐regulated modules. All gene modules were identified using Monocle332 Note that CXCL14 and ADAMDEC1 were the top two markers of module1 (arrowed), and are primarily expressed in sub‐cluster2. [file JCMM-28-e18344-s003.jpg]
