# Supplementary material for: Single cell sequencing data identify distinct B cell and fibroblast populations in stricturing Crohn's disease
Source: J Cell Mol Med. 2024 Apr 29;28(9):e18344. doi: 10.1111/jcmm.18344 (PMC11058334; doi:10.1111/jcmm.18344)
Supplement: Supplementary file 4 — Table S1. Gene markers that define fibroblast cell clusters C9 and C12. List of all differentially expressed genes identified using the findmarkers function (MAST algorithm) to compare C9 to C12 fibroblast populations. The table was created using a filtering fold change of >1.5 and adjusted p‐values of <0.01. Each table has been ordered on pct.1 and then by average fold change. Genes with known involvement in the fibrotic process and mentioned in the text (collagen genes or potential markers of fibrosis) are highlighted in bold. Whilst these genes are not at the very top of Table S1, they are highly significant (p‐value adjusted <9.0E‐05). [file JCMM-28-e18344-s001.docx]

**Table S1. Gene markers that define fibroblast cell clusters C9 and C12.** List of all differentially expressed genes identified using the findmarkers function (MAST algorithm) to compare C9 to C12 fibroblast populations. The table was created using a filtering fold change of >1.5 and adjusted p-values of < 0.01). Each table has been ordered on pct.1 and then by average fold change. Genes with known involvement in the fibrotic process and mentioned in the text (collagen genes or potential markers of fibrosis) are highlighted in bold. Whilst these genes are not at the very top of Table S1, they are highly significant (p value adjusted<9.0E-05).

**C12 compared to C9**

| Gene | p_val | avg_log2FC | pct.1 | pct.2 | p_val_adj |
| --- | --- | --- | --- | --- | --- |
| **DCN** | 5.52E-39 | 0.727796 | 1 | 0.949 | 1.4E-34 |
| CFD | 1.2E-54 | 1.112183 | 0.997 | 0.858 | 3.03E-50 |
| TMSB4X | 9.7E-36 | 0.781292 | 0.997 | 0.961 | 2.45E-31 |
| CST3 | 2.02E-47 | 0.734969 | 0.997 | 0.922 | 5.11E-43 |
| B2M | 6.59E-15 | 0.627643 | 0.997 | 0.986 | 1.67E-10 |
| IGFBP6 | 9.5E-128 | 2.058368 | 0.995 | 0.748 | 2.4E-123 |
| PLAC9 | 3E-123 | 1.766458 | 0.995 | 0.638 | 7.6E-119 |
| S100A10 | 2.2E-110 | 1.583371 | 0.995 | 0.757 | 5.7E-106 |
| HLA-B | 1.07E-19 | 0.648267 | 0.995 | 0.942 | 2.71E-15 |
| FBN1 | 2.4E-120 | 2.136493 | 0.992 | 0.792 | 6E-116 |
| S100A6 | 3.48E-66 | 1.047138 | 0.992 | 0.938 | 8.79E-62 |
| GSN | 2.29E-38 | 0.769769 | 0.992 | 0.883 | 5.8E-34 |
| TIMP1 | 2.42E-57 | 1.078245 | 0.99 | 0.852 | 6.11E-53 |
| TIMP2 | 9.52E-31 | 0.611803 | 0.99 | 0.863 | 2.41E-26 |
| TNXB | 7.22E-82 | 1.421838 | 0.987 | 0.76 | 1.82E-77 |
| S100A4 | 3.38E-83 | 1.312589 | 0.987 | 0.815 | 8.56E-79 |
| TIMP3 | 1.81E-63 | 1.175705 | 0.975 | 0.711 | 4.59E-59 |
| FSTL1 | 5.46E-71 | 1.470796 | 0.97 | 0.747 | 1.38E-66 |
| ANXA2 | 2.01E-55 | 0.951132 | 0.97 | 0.776 | 5.08E-51 |
| LTBP4 | 3.56E-47 | 0.885592 | 0.97 | 0.77 | 9E-43 |
| MFAP5 | 8.3E-162 | 2.944869 | 0.957 | 0.297 | 2.1E-157 |
| SPARC | 2.14E-18 | 0.620161 | 0.957 | 0.81 | 5.42E-14 |
| EFEMP1 | 5.63E-49 | 0.952892 | 0.952 | 0.698 | 1.42E-44 |
| CD248 | 9.05E-95 | 1.524008 | 0.95 | 0.477 | 2.29E-90 |
| S100A11 | 1.69E-40 | 0.893673 | 0.947 | 0.742 | 4.26E-36 |
| IGFBP5 | 1.04E-16 | 0.734037 | 0.942 | 0.779 | 2.64E-12 |
| FN1 | 4.11E-93 | 2.09181 | 0.92 | 0.417 | 1.04E-88 |
| EMP3 | 1.06E-56 | 1.192682 | 0.92 | 0.653 | 2.67E-52 |
| CD99 | 5.59E-30 | 0.653976 | 0.915 | 0.751 | 1.41E-25 |
| TXNIP | 5.17E-18 | 0.619232 | 0.915 | 0.748 | 1.31E-13 |
| FBLN2 | 3.51E-84 | 1.493094 | 0.912 | 0.386 | 8.88E-80 |
| GPX3 | 2.46E-32 | 0.748008 | 0.91 | 0.631 | 6.21E-28 |
| TAGLN2 | 1.48E-25 | 0.590009 | 0.905 | 0.661 | 3.74E-21 |
| SEMA3C | 3E-139 | 2.39509 | 0.894 | 0.179 | 7.5E-135 |
| CD55 | 8.5E-116 | 2.439183 | 0.889 | 0.333 | 2.1E-111 |
| PCOLCE | 3.05E-19 | 0.614978 | 0.879 | 0.672 | 7.72E-15 |
| SFRP1 | 1.5E-23 | 0.698172 | 0.877 | 0.602 | 3.8E-19 |
| RHOA | 1.23E-27 | 0.69305 | 0.869 | 0.652 | 3.11E-23 |
| RNH1 | 1.11E-25 | 0.620559 | 0.867 | 0.625 | 2.81E-21 |
| CPQ | 5.83E-30 | 0.638886 | 0.862 | 0.572 | 1.47E-25 |
| SDC2 | 7.94E-44 | 0.883742 | 0.859 | 0.465 | 2.01E-39 |
| CLU | 6.32E-34 | 0.819867 | 0.854 | 0.502 | 1.6E-29 |
| SCARA5 | 1.9E-106 | 1.820405 | 0.849 | 0.205 | 4.9E-102 |
| SH3BGRL3 | 8.67E-56 | 1.267391 | 0.847 | 0.513 | 2.19E-51 |
| LDHA | 2.64E-21 | 0.666494 | 0.839 | 0.614 | 6.67E-17 |
| C1QTNF3 | 3.25E-34 | 0.726573 | 0.827 | 0.451 | 8.21E-30 |
| MT2A | 1.92E-11 | 1.002204 | 0.824 | 0.636 | 4.85E-07 |
| PLA2G2A | 1.96E-56 | 1.757877 | 0.822 | 0.348 | 4.95E-52 |
| CLIC1 | 9.11E-21 | 0.610611 | 0.819 | 0.593 | 2.3E-16 |
| UAP1 | 5.87E-73 | 1.5602 | 0.809 | 0.305 | 1.48E-68 |
| VKORC1 | 2.64E-21 | 0.598223 | 0.804 | 0.591 | 6.67E-17 |
| PPIC | 1.21E-47 | 1.074767 | 0.796 | 0.437 | 3.06E-43 |
| SMIM14 | 1.67E-26 | 0.63365 | 0.779 | 0.456 | 4.21E-22 |
| ADAMTS5 | 2.71E-80 | 1.992449 | 0.774 | 0.23 | 6.84E-76 |
| PIGT | 4.54E-39 | 0.843917 | 0.771 | 0.393 | 1.15E-34 |
| CADM3 | 2.98E-72 | 1.416784 | 0.769 | 0.232 | 7.53E-68 |
| HTRA3 | 1.25E-34 | 1.215896 | 0.766 | 0.415 | 3.17E-30 |
| C17orf58 | 8.71E-81 | 1.80359 | 0.764 | 0.226 | 2.2E-76 |
| CLEC3B | 1E-114 | 1.844727 | 0.759 | 0.1 | 2.6E-110 |
| KLF2 | 2.29E-13 | 0.732776 | 0.746 | 0.572 | 5.78E-09 |
| CLTB | 2.38E-30 | 0.795704 | 0.744 | 0.417 | 6.01E-26 |
| MEDAG | 1.4E-34 | 0.795629 | 0.741 | 0.353 | 3.55E-30 |
| ABLIM1 | 2.51E-42 | 0.871546 | 0.739 | 0.309 | 6.35E-38 |
| CD34 | 1.3E-38 | 0.858977 | 0.739 | 0.336 | 3.29E-34 |
| TPPP3 | 2.77E-67 | 1.562293 | 0.734 | 0.232 | 7.01E-63 |
| GAS7 | 3.09E-44 | 1.041554 | 0.734 | 0.331 | 7.82E-40 |
| REXO2 | 6.31E-18 | 0.597582 | 0.731 | 0.496 | 1.6E-13 |
| ACKR3 | 3.69E-73 | 1.557218 | 0.724 | 0.179 | 9.34E-69 |
| CREB5 | 3E-47 | 1.099362 | 0.721 | 0.281 | 7.59E-43 |
| TRIOBP | 5.68E-30 | 0.670557 | 0.721 | 0.365 | 1.44E-25 |
| METRNL | 1.11E-63 | 1.259878 | 0.714 | 0.227 | 2.81E-59 |
| PTGIS | 1.95E-37 | 0.903776 | 0.711 | 0.313 | 4.93E-33 |
| DBN1 | 8.1E-69 | 1.243779 | 0.686 | 0.17 | 2.05E-64 |
| SERPINE2 | 1.47E-19 | 0.887899 | 0.686 | 0.4 | 3.72E-15 |
| ADI1 | 5.91E-26 | 0.739161 | 0.686 | 0.376 | 1.5E-21 |
| ITM2A | 3.27E-41 | 1.135903 | 0.678 | 0.291 | 8.26E-37 |
| ZNF385A | 3.06E-49 | 1.031576 | 0.673 | 0.229 | 7.73E-45 |
| ARHGAP29 | 2.77E-46 | 0.941716 | 0.666 | 0.23 | 7.01E-42 |
| UGP2 | 2.22E-22 | 0.763663 | 0.653 | 0.381 | 5.62E-18 |
| OSR2 | 3.58E-29 | 0.757793 | 0.651 | 0.292 | 9.04E-25 |
| CRIP1 | 6.17E-29 | 1.217787 | 0.641 | 0.302 | 1.56E-24 |
| YWHAH | 4.97E-25 | 0.772146 | 0.631 | 0.316 | 1.26E-20 |
| PRSS23 | 5.89E-59 | 1.259388 | 0.628 | 0.156 | 1.49E-54 |
| AXL | 5.19E-25 | 0.67752 | 0.621 | 0.305 | 1.31E-20 |
| F10 | 1.33E-19 | 0.666597 | 0.621 | 0.336 | 3.36E-15 |
| PHGDH | 1.03E-38 | 0.755862 | 0.613 | 0.21 | 2.6E-34 |
| SLPI | 5.68E-52 | 1.76331 | 0.595 | 0.16 | 1.44E-47 |
| PI16 | 3.24E-68 | 1.864965 | 0.59 | 0.09 | 8.2E-64 |
| PCOLCE2 | 4.2E-79 | 1.823331 | 0.59 | 0.072 | 1.06E-74 |
| HEG1 | 1.03E-29 | 0.589509 | 0.583 | 0.23 | 2.6E-25 |
| PPP1R14B | 2.09E-23 | 0.751395 | 0.575 | 0.292 | 5.28E-19 |
| LOXL1 | 7.85E-38 | 1.005406 | 0.573 | 0.199 | 1.99E-33 |
| CLDN11 | 6.2E-29 | 0.634452 | 0.573 | 0.226 | 1.57E-24 |
| LRRC17 | 5.3E-49 | 1.05657 | 0.568 | 0.134 | 1.34E-44 |
| SEMA3E | 1.92E-67 | 1.371876 | 0.555 | 0.072 | 4.86E-63 |
| UGDH | 3.35E-32 | 0.72396 | 0.553 | 0.194 | 8.47E-28 |
| LINC01133 | 2.04E-52 | 1.01731 | 0.535 | 0.104 | 5.17E-48 |
| ADAMTSL4 | 7.8E-38 | 0.693549 | 0.535 | 0.152 | 1.97E-33 |
| EMILIN2 | 6.67E-47 | 0.846781 | 0.528 | 0.114 | 1.69E-42 |
| GFPT2 | 4.76E-29 | 0.774514 | 0.513 | 0.179 | 1.2E-24 |
| VASN | 1.6E-27 | 0.714992 | 0.508 | 0.182 | 4.04E-23 |
| PROCR | 1.21E-45 | 1.105508 | 0.492 | 0.1 | 3.07E-41 |
| TBC1D12 | 5.34E-27 | 0.652958 | 0.492 | 0.173 | 1.35E-22 |
| ITGA11 | 5.87E-34 | 0.789623 | 0.48 | 0.131 | 1.48E-29 |
| SEMA3B | 1.33E-37 | 0.743557 | 0.477 | 0.115 | 3.37E-33 |
| CYTOR | 3.67E-19 | 0.724797 | 0.467 | 0.201 | 9.28E-15 |
| TRIO | 1.12E-21 | 0.727835 | 0.455 | 0.179 | 2.84E-17 |
| HSD3B7 | 4.39E-39 | 0.683122 | 0.437 | 0.086 | 1.11E-34 |
| C12orf75 | 1.26E-36 | 0.711187 | 0.435 | 0.095 | 3.18E-32 |
| SHISA3 | 4.96E-19 | 0.638221 | 0.417 | 0.159 | 1.25E-14 |
| GALNT15 | 2.31E-39 | 0.891362 | 0.384 | 0.058 | 5.85E-35 |
| AIF1L | 5.59E-46 | 0.696697 | 0.384 | 0.04 | 1.41E-41 |
| TNFAIP6 | 3.15E-07 | 0.69611 | 0.379 | 0.23 | 0.007957 |
| GALNT12 | 1.29E-32 | 0.653758 | 0.372 | 0.07 | 3.25E-28 |
| NTM | 1.23E-27 | 0.597367 | 0.334 | 0.067 | 3.11E-23 |
| DPP4 | 3.63E-25 | 0.593136 | 0.329 | 0.076 | 9.18E-21 |
| TRAC | 3.14E-30 | 0.670273 | 0.279 | 0.034 | 7.95E-26 |
| ADAMTS16 | 8.99E-30 | 0.636292 | 0.259 | 0.026 | 2.27E-25 |
| CD24 | 3.4E-19 | 0.772708 | 0.239 | 0.048 | 8.6E-15 |
| PRG4 | 2.2E-09 | 0.642053 | 0.048 | 0 | 5.57E-05 |

**C9 compared to C12**

|  | p_val | avg_log2FC | pct.1 | pct.2 | p_val_adj |
| --- | --- | --- | --- | --- | --- |
| **JUN** | 2.35E-23 | 0.891514 | 0.947 | 0.962 | 5.94E-19 |
| EGR1 | 2.56E-09 | 0.660965 | 0.908 | 0.894 | 6.47E-05 |
| CALD1 | 1.09E-33 | 0.65643 | 0.9 | 0.965 | 2.75E-29 |
| SPARCL1 | 3.85E-35 | 0.883563 | 0.897 | 0.905 | 9.73E-31 |
| FOSB | 1.13E-26 | 0.961446 | 0.885 | 0.749 | 2.87E-22 |
| CXCL12 | 1.17E-32 | 0.943173 | 0.876 | 0.789 | 2.95E-28 |
| **COL6A3** | 7E-18 | 0.652227 | 0.857 | 0.872 | 1.77E-13 |
| DPT | 1.48E-16 | 0.847522 | 0.788 | 0.714 | 3.74E-12 |
| SAT1 | 1.09E-09 | 0.753806 | 0.737 | 0.739 | 2.75E-05 |
| SOD2 | 4.78E-18 | 1.170726 | 0.72 | 0.643 | 1.21E-13 |
| PPP1R15A | 4.15E-13 | 0.659816 | 0.709 | 0.651 | 1.05E-08 |
| PTGDS | 1.56E-26 | 1.483627 | 0.687 | 0.394 | 3.94E-22 |
| APOE | 1.8E-09 | 0.727634 | 0.687 | 0.565 | 4.55E-05 |
| BTG1 | 1.67E-18 | 0.846339 | 0.683 | 0.595 | 4.22E-14 |
| IGFBP7 | 5.6E-12 | 0.680825 | 0.683 | 0.764 | 1.42E-07 |
| LHFPL6 | 3.54E-17 | 0.599089 | 0.67 | 0.686 | 8.96E-13 |
| C7 | 1.33E-51 | 2.38598 | 0.669 | 0.239 | 3.36E-47 |
| A2M | 4.76E-27 | 1.169691 | 0.663 | 0.568 | 1.2E-22 |
| IGF1 | 2.81E-21 | 1.023047 | 0.644 | 0.497 | 7.1E-17 |
| TPM2 | 1.36E-40 | 1.432498 | 0.641 | 0.41 | 3.44E-36 |
| RSPO3 | 5.45E-22 | 0.635414 | 0.636 | 0.671 | 1.38E-17 |
| **LAMB1** | 3.91E-13 | 0.604513 | 0.599 | 0.548 | 9.9E-09 |
| **GREM1** | 8.39E-15 | 1.107837 | 0.565 | 0.344 | 2.12E-10 |
| CRISPLD2 | 3.11E-14 | 0.694896 | 0.561 | 0.47 | 7.87E-10 |
| EMILIN1 | 6.38E-27 | 1.032786 | 0.547 | 0.327 | 1.61E-22 |
| NEXN | 2E-13 | 0.592071 | 0.519 | 0.44 | 5.06E-09 |
| TSC22D1 | 2.49E-11 | 0.700893 | 0.51 | 0.42 | 6.29E-07 |
| ADAMTS1 | 1.6E-08 | 0.855066 | 0.505 | 0.558 | 0.000404 |
| CD302 | 9.79E-14 | 0.598082 | 0.482 | 0.415 | 2.47E-09 |
| TAGLN | 1.36E-15 | 1.628015 | 0.471 | 0.324 | 3.45E-11 |
| MDK | 4.3E-11 | 0.642444 | 0.471 | 0.291 | 1.09E-06 |
| HSPB6 | 4.86E-19 | 0.680471 | 0.465 | 0.352 | 1.23E-14 |
| RBP1 | 2.19E-19 | 0.834128 | 0.451 | 0.209 | 5.55E-15 |
| **COL4A2** | 8.03E-24 | 0.940064 | 0.449 | 0.191 | 2.03E-19 |
| GBP1 | 8.72E-08 | 0.789271 | 0.431 | 0.362 | 0.002206 |
| CCL2 | 1.53E-13 | 1.659622 | 0.428 | 0.234 | 3.87E-09 |
| **COL18A1** | 1.92E-15 | 0.889494 | 0.425 | 0.286 | 4.84E-11 |
| PHLDA1 | 2.65E-08 | 0.71377 | 0.404 | 0.322 | 0.00067 |
| CYP7B1 | 1.93E-13 | 0.64462 | 0.376 | 0.188 | 4.89E-09 |
| NDRG2 | 2.46E-20 | 0.737922 | 0.369 | 0.143 | 6.22E-16 |
| CYGB | 7.79E-23 | 0.783972 | 0.364 | 0.133 | 1.97E-18 |
| **COL4A1** | 1.78E-18 | 0.92709 | 0.359 | 0.138 | 4.5E-14 |
| GAS6 | 1.41E-17 | 0.711539 | 0.355 | 0.168 | 3.56E-13 |
| NR2F1 | 3.07E-21 | 0.860581 | 0.353 | 0.131 | 7.77E-17 |
| PRKAR2B | 8.43E-11 | 0.614476 | 0.35 | 0.204 | 2.13E-06 |
| PLEKHH2 | 1.91E-18 | 0.88314 | 0.345 | 0.158 | 4.82E-14 |
| GUCY1A1 | 1.89E-13 | 0.749041 | 0.344 | 0.204 | 4.79E-09 |
| MYLK | 6.07E-23 | 1.193682 | 0.341 | 0.113 | 1.53E-18 |
| SPON1 | 8.76E-12 | 0.594951 | 0.331 | 0.158 | 2.22E-07 |
| VCAM1 | 6.7E-14 | 0.675541 | 0.325 | 0.166 | 1.7E-09 |
| RAMP1 | 1.53E-22 | 1.155701 | 0.316 | 0.103 | 3.88E-18 |
| CDH11 | 5.64E-13 | 0.624799 | 0.311 | 0.141 | 1.43E-08 |
| CCL11 | 2.65E-31 | 2.678723 | 0.309 | 0.04 | 6.71E-27 |
| CHCHD10 | 1.71E-16 | 0.612831 | 0.303 | 0.103 | 4.32E-12 |
| KCNN3 | 8.18E-12 | 1.016196 | 0.303 | 0.148 | 2.07E-07 |
| GGT5 | 2.03E-20 | 0.887518 | 0.299 | 0.075 | 5.13E-16 |
| **ACTA2** | 3.76E-09 | 1.009069 | 0.297 | 0.226 | 9.5E-05 |
| CTSC | 4.77E-14 | 1.18124 | 0.291 | 0.133 | 1.21E-09 |
| LMOD1 | 1.99E-15 | 0.624765 | 0.281 | 0.098 | 5.03E-11 |
| CCN2 | 6E-11 | 1.064428 | 0.272 | 0.291 | 1.52E-06 |
| ACTN1 | 7.23E-28 | 0.819493 | 0.269 | 0.033 | 1.83E-23 |
| TDO2 | 1.17E-22 | 0.746097 | 0.243 | 0.035 | 2.95E-18 |
| CARMN | 1.64E-19 | 0.737473 | 0.218 | 0.03 | 4.14E-15 |
| FMO2 | 4.46E-17 | 0.862659 | 0.212 | 0.043 | 1.13E-12 |
| FRZB | 1.04E-19 | 0.704692 | 0.201 | 0.023 | 2.63E-15 |
| TNC | 5.38E-17 | 1.00923 | 0.188 | 0.028 | 1.36E-12 |
| **COL15A1** | 2.8E-17 | 0.606153 | 0.182 | 0.023 | 7.07E-13 |
| CXCL14 | 6.69E-11 | 2.873001 | 0.176 | 0.07 | 1.69E-06 |
| SCN7A | 2.1E-09 | 0.736626 | 0.168 | 0.058 | 5.3E-05 |
| **ADAMDEC1** | 2.2E-15 | 3.185464 | 0.166 | 0.023 | 5.55E-11 |
| IGFBP3 | 2.97E-11 | 0.585254 | 0.135 | 0.176 | 7.52E-07 |
| IGFBP2 | 1.11E-10 | 0.588831 | 0.117 | 0.02 | 2.81E-06 |
| CHI3L1 | 4.08E-11 | 1.524184 | 0.107 | 0.01 | 1.03E-06 |
| CCL19 | 7.72E-11 | 1.652495 | 0.068 | 0 | 1.95E-06 |
